# Skeletal muscle index combined with IgM predicts prognosis in gastric cancer patients who underwent surgery

**DOI:** 10.3389/fimmu.2025.1633926

**Published:** 2025-11-21

**Authors:** Yunxin Xu, Yue Xing, Zhongze Du, Ruihu Zhao, Guiming Deng, Haibin Song, Yingwei Xue, Hongjiang Song

**Affiliations:** Department of Gastrointestinal Surgery, Harbin Medical University Cancer Hospital, Harbin Medical University, Harbin, Heilongjiang, China

**Keywords:** skeletal muscle index, IgM, gastric cancer, surgery, prognosis

## Abstract

**Background:**

The purpose of this study is to investigate the combination of skeletal muscle index (SMI) and immunoglobulin M (IgM) to form the SMI-IgM score to predict the prognosis of patients who underwent surgery for gastric cancer.

**Patients and methods:**

In this study, 190 patients operated for gastric cancer from July 2016 to December 2017 were collected. According to the optimal critical values of skeletal muscle index and immunoglobulin M, all patients were divided into three groups. We used Kaplan-Meier survival curves and log-rank test to assess the differences in progression-free survival time (PFS) and overall survival time (OS) between the 3 groups of patients. Independent predictors were identified using Cox regression, and nomogram plots were produced to predict confounded 1-, 3-, and 5-year survival rates based on independent predictors.

**Results:**

There were 68 patients (35.8%) in the SMI-IgM 1 group, 85 patients (44.7%) in the SMI-IgM 2 group, and 37 patients (19.5%) in the SMI-IgM 3 group. Patients in the SMI-IgM 1 group had worse PFS (HR = 0.345, 95% CI: 0.226-0.525, *p*** < **0.001) and OS (HR = 0.345, 95% CI: 0.227-0.525, *p*** < **0.001). The multifactorial analysis showed that SMI-IgM score was an independent predictor of PFS and OS in patients. The calibration curves show better predictive efficacy of the column charts in years 3 and 5.

**Conclusion:**

The SMI-IgM score could well respond to the nutritional and immune status of the body, and could be used as a new predictor for patients undergoing surgery for gastric cancer.

## Introduction

According to the statistical data of World Health Organisation, gastric cancer is the fifth most common cancer in the world and the fourth leading cause of cancer deaths (1). Radical gastrectomy is still the mainstay of treatment for gastric cancer at present (2). However, many gastric cancer patients still have recurrence and distant metastases after undergoing surgery (3, 4). Therefore, a new biomarker is urgently needed to accurately predict the prognosis of gastric cancer patients.

Cachexia is an internationally recognized independent prognostic factor affecting patients with cancer. Some studies have shown that 50%-80% of cancer patients suffer from cachexia and it contributes to the deaths of 20%-40% of cancer patients (5–7). Some researchers have defined cancer cachexia as a loss of more than 2% of body weight in patients with malignant tumors and associated sarcopenia (8). In 2016, sarcopenia was recognized as a separate disease (9). Sarcopenia is a skeletal muscle disease characterized by progressive loss of muscle mass and function, manifested by low muscle strength and reduced muscle quantity or quality (10, 11). It also has a high prevalence among cancer patients (12). Numerous studies have shown that sarcopenia is one of the predictors of poor prognosis for surgical complications and overall survival in patients with various solid tumors (13–15). Skeletal muscle index (SMI) is an important parameter for measuring body composition, and it is obtained by quantifying skeletal muscle on computed tomography (CT) scans based on patient height (16, 17). Low SMI is an important manifestation of sarcopenia (18). In addition, the inflammatory state of the body can affect the prognosis of tumor patients. Some studies have shown that lymphocytes (L) and C-reactive protein (CRP) are associated with lower survival rates in gastric cancer (19). Immunoglobulin M (IgM) acts primarily as an early immune response following antigenic stimulation and it is associated with recurrence and metastasis of many tumors, including gastric cancer (20–22). Overall, patients with sarcopenia and those in an inflammatory state have a poor prognosis.

Numerous studies have shown that skeletal muscle index can predict poor prognosis in gastric cancer (23–25). However, no article has reported that the SMI-IgM score, a combined indicator of SMI and IgM, predicts effectiveness in patients underwent surgery for gastric cancer. In this study, we aimed to evaluate the predictive efficacy of the SMI-IgM score in gastric cancer patients who underwent surgery.

## Materials and methods

### Patients

We continuously collected 190 gastric cancer patients who underwent surgical treatment at Harbin Medical University Cancer Hospital from July 2016 to December 2017. Due to the retrospective nature of this study, the Ethics Committee of Harbin Medical University Cancer Hospital waived the requirement for informed consent (Ethics Number: 2019-57-IIT). We conducted statistical analysis on clinical information, laboratory tests, and pathological data of 190 patients based on the Helsinki Declaration and its amendments. The inclusion criteria are: (1) All patients are gastric cancer patients who have undergone surgical treatment; (2) All patients underwent specific protein testing; (3) All patients underwent abdominal computed tomography (CT) scans at the Cancer Hospital of Harbin Medical University. The exclusion criteria are: (1) Patients with chronic diseases; (2) The patient’s body is in an acute inflammatory state; (3) Patients with gastric cancer combined with other primary malignant tumors; (4) The patient has no complete clinical data.

### Data collection

Patients were followed up through telephone or outpatient services, with a follow-up every 3–6 months for the first two years, every 6–12 months for the third to fifth years, and annually thereafter. Progression free survival (PFS) is defined as the time period from the first day of surgery to the date of disease progression, withdrawal from follow-up, or last follow-up. The evidence of progress is mainly obtained through chest and abdominal X-rays or CT scans. The overall survival (OS) is defined as the time interval from the date of surgery to the date of death, the date of withdrawal from follow-up, or the last follow-up. We use the hospital electronic medical record system to obtain clinical and pathological information of patients.

### The assessment of SMI and patients group

All abdominal CT images were analyzed using 3D Slicer (version 4.10.2, www.slicer.org) by radiologists from Harbin Medical University Cancer Hospital. All physicians have worked in the radiology department for more than 10 years. We measure the skeletal muscle area (cm^2^) at the level of the third lumbar vertebra (L3), subcutaneous fat area (SAT), and visceral fat area (VAT). The Hounsfield unit threshold for skeletal muscle is set to -29 to 150, and the Hounsfield unit threshold for fat is set to -190 to -30. The definition of SMI for L3 is: skeletal muscle area (cm^2^)/Height square (m^2^). The level of peripheral specific proteins were measured and analyzed using a specific protein analyzer (IMMAGE800). Specific proteins include immunoglobulin A (IgA), immunoglobulin G (IgG), IgM, light chain immunoglobulin (KAP), heavy chain immunoglobulin (LAM), and KAP/LAM.

**Figure 1:**
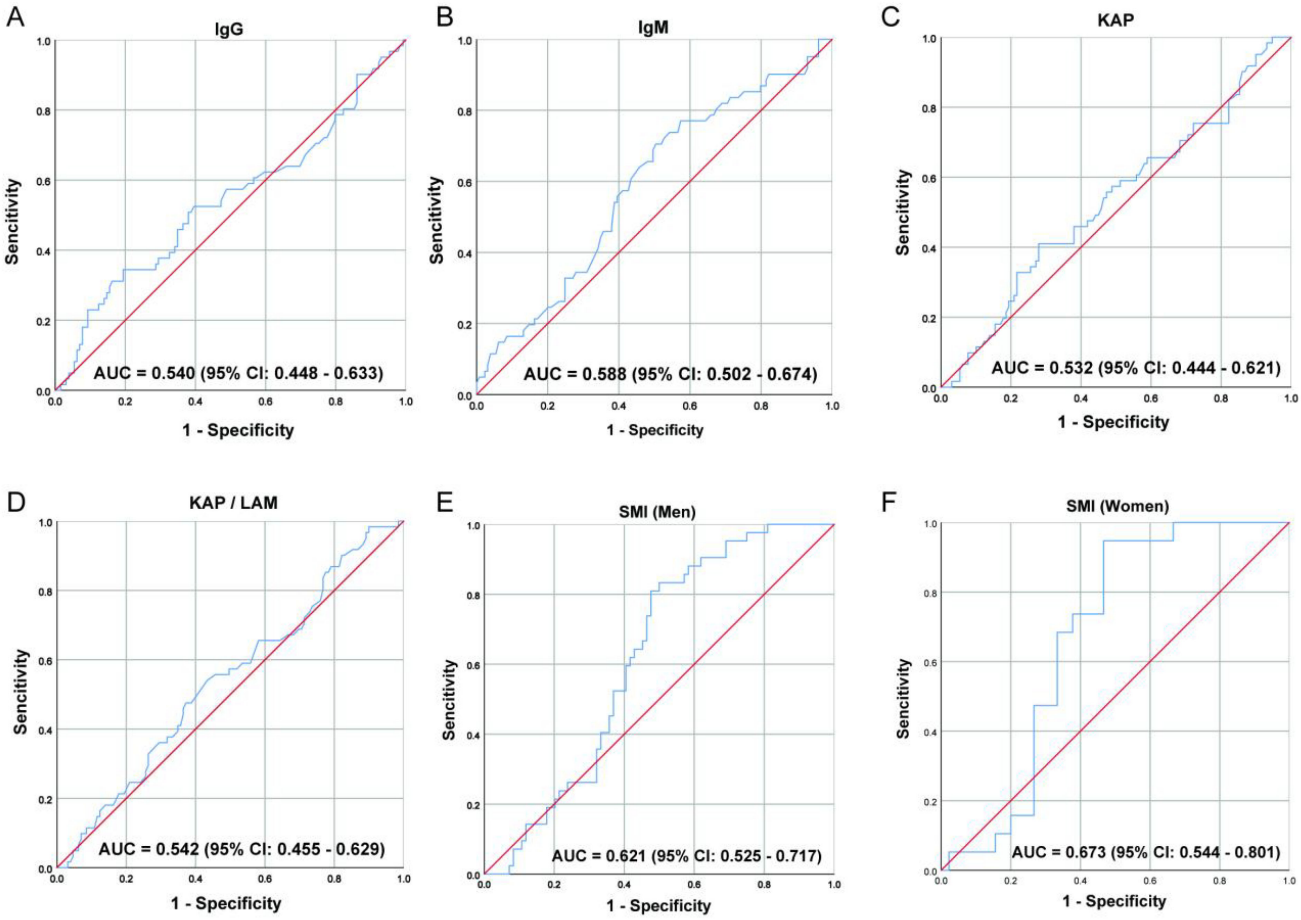
The ROC curve of **(A)** IgG, **(B)** IgM, **(C)** KAP, **(D)** KAP/LAM, **(E)** SMI (Men), and **(F)** SMI (Women).

The optimal cutoff values for SMI and IgM are obtained using the maximum Youden index calculated from the receiver operating characteristic (ROC) curve. The optimal cut-off values of SMI and IgM with the highest Youden index were obtained. The optimal cut-off value of IgM was 0.93 g/L (**Figure 1B**). The optimal cut-off value for SMI is 39.26 cm²/m² (**Figure 1E**) for males and 31.41 cm²/m² (**Figure 1F**) for females. According to the optimal cut-off values of SMI and IgM, all patients were divided into three groups: SMI-IgM score 3 (n = 68): high IgM (≥ 0.93 g/L) and high SMI (men ≥ 39.26 cm²/m², women ≥ 31.41 cm²/m²); SMI-IgM score 2 (n = 85): high IgM (≥ 0.93 g/L) and low SMI (men < 39.26 cm²/m², women < 31.41 cm²/m²), or low IgM (< 0.93 g/L) and high SMI (men ≥ 39.26 cm²/m², women ≥ 31.41 cm²/m²); SMI-IgM score 1(n = 37): low IgM (< 0.93 g/L) and low SMI (men < 39.26 cm²/m², women < 31.41 cm²/m²).

### Statistical analysis

We use mean with standard deviation (SD) or median with interquartile range (IQR) to represent continuous variables. Use percentages to represent categorical variables. We compared the differences between continuous variables using one-way ANOVA and Kruskal-Wallis rank-sum test. The differences between categorical variables are compared using chi square test or Fisher’s exact test. We used Kaplan-Meier survival curve and log-rank test to calculate the difference in survival rate and survival time. Univariate and multivariate analyses were conducted using the Cox proportional risk model. Variables with *p***< **0.05 in univariate analysis were input into multivariate Cox regression analysis. We further evaluated potential multicollinearity by calculating variance inflation factors (VIFs). We evaluate relative risk through hazard ratio (HR) and 95% confidence interval (CI). We constructed a nomogram to predict the 1-year, 3-year, and 5-year survival probabilities of PFS and OS. Calibration curve analysis is used to evaluate the prognostic predictive ability of nomograms. Finally, there is a statistically significant difference in *p* values < 0.05 between the two sides.

## Results

### Patient characteristics

This study continuously enrolled 190 patients, with a median age of 60 years old, consisting of 126 males (66.3%) and 64 females (33.7%). We found through chi square test or Fisher’s exact test that the SMI-IgM score was associated with Melena (*p* = 0.042), weight loss (*p* = 0.026), tumor size (p = 0.001), and pTNM staging (*p* = 0.003). The one-way ANOVA and Kruskal-Wails rank sum test showed that SMI-IgM score was related to age, body mass index (BMI), and SAT (all *p*** < **0.05). **Table 1** shows the clinical characteristics of patients grouped by SMI-IgM score.

**Table 1:** Clinical, pathological and laboratory information of all patients.

n	Level	SMI-IgM score 1	SMI-IgM score 2	SMI-IgM score 3	P
		68	85	37	
Age	median (IQR)	63.00 (56.00-68.75)	60.00 (51.00-66.5)	52.00(44.50-62.50)	<0.001
BMI	median (IQR)	20.51 (18.39-23.55)	22.49 (20.80-24.81)	23.44(21.38-25.39)	<0.001
Sex	male	43(63.2)	64(75.3)	19(51.4)	0.029
	female	25(36.8)	21(24.7)	18(48.6)	
Stomachache	no	13(19.1)	24(12.6)	11(29.7)	0.341
	yes	55(80.9)	61(71.8)	26(70.3)	
Melaena	no	48(70.6)	66(77.6)	34(91.9)	0.042
	yes	20(29.4)	9(22.4)	3(8.1)	
Weight loss	no	22(32.4)	46(54.1)	17(45.9)	0.026
	yes	46(67.6)	39(45.9)	20(54.1)	
Tumor size	<50 mm	25(36.8)	45(52.9)	28(75.7)	0.001
	≥50 mm	43(63.2)	40(47.1)	9(24.3	
pTNM	Tis/0 + I + II	36(52.9)	56(65.9)	32(86.5)	0.003
	III + IV	32(35.8)	29(43.9)	5(13.5)	
ALT (U/L)	mean ± SD	17.95 ± 8.64	21.66 ± 16.01	20.59 ± 8.36	0.154
LDH (U/L)	median (IQR)	163.00 (147.00-183.00)	161.00 (142.00-179.50)	156.00(144.00-175.50)	0.467
TBIL (μmol/L)	mean ± SD	12.17 ± 9.55	12.49 ± 5.16	13.12 ± 5.15	0.805
TP (g/L)	median (IQR)	67.00 (61.00-70.50)	67.1 (65.00-72.00)	72.00(67.00-76.00)	0.001
ALB (g/L)	mean ± SD	39.10 ± 4.53	40.64 ± 3.76	41.23 ± 4.23	0.020
PALB (mg/L)	mean ± SD	246.87 ± 63.08	274.47 ± 75.06	279.68 ± 79.82	0.028
UREA (mmol/L)	mean ± SD	5.92 ± 1.80	6.65 ± 2.13	5.24 ± 1.60	0.422
WBC (109/L)	mean ± SD	6.56 ± 1.90	6.65 ± 2.47	6.93 ± 2.40	0.725
NEU (109/L)	mean ± SD	4.01 ± 2.90	4.06 ± 2.47	4.06 ± 2.47	0.994
L (109/L)	mean ± SD	1.89 ± 0.68	1.90 ± 0.71	2.10 ± 0.64	0.034
Mono (109/L)	mean ± SD	0.50 ± 0.22	0.48 ± 0.19	0.44 ± 0.15	0.449
RBC (1012/L)	mean ± SD	4.18 ± 0.61	4.42 ± 0.55	4.63 ± 0.46	<0.001
P (109/L)	mean ± SD	274.34 ± 86.83	248.98 ± 74.59	247.60 ± 54.77	0.148
CEA (ng/ml)	median (IQR)	2.53 (1.23-5.80)	1.82 (1.02-2.99)	1.94(1.01-2.40)	0.042
CA724 (U/mL)	median (IQR)	8.66 (4.02-23.10)	8.87 (5.48-17.81)	9.46(5.07-12.92)	0.785
CA199 (U/mL)	median (IQR)	2.85 (1.18-6.94)	1.75 (1.09-3.89)	2.19(0.99-5.18)	0.336
CA125 (U/mL)	median (IQR)	11.00 (7.71-17.40)	8.98 (6.80-12.28)	9.33(7.21-13.89)	0.077
IgA (g/L)	median (IQR)	2.15 (1.35-2.79)	2.15 (1.62-2.88)	2.48(2.48(1.65-3.19)	0.153
IgG (g/L)	mean ± SD	10.26 ± 2.99	10.79 ± 2.63	12.43 ± 1.27	0.002
KAP (g/L)	median (IQR)	7.97 (6.23-9.34)	8.31 (7.06-9.77)	9.52(8.01-11.90)	0.003
LAM (g/L)	median (IQR)	4.72 (4.03-5.67)	4.59 (4.04-5.47)	5.33(4.55-6.04)	0.062
KAP/LAM	median (IQR)	1.74 (1.43-1.97)	1.81 (1.63-2.00)	1.81(1.60-2.23)	0.056
SAT (cm²)	median (IQR)	66.43 (39.07-105.77)	80.11 (52.77-119.82)	107.39(73.54-149.92)	0.001
VAT (cm²)	median (IQR)	57.15 (20.84-86.03)	70.36 (30.83-103.41)	76.97(36.19-118.42)	0.073

SMI, skeletal muscle index; IgM, immunoglobulin M; SD, standard deviation; IQR, interquartile range; BMI, body mass index; ALT, alanine aminotransferase; LDH, lactate dehydrogenase; TBIL, total bilirubin; TP, total protein; ALB, albumin; PALB, prealbumin; WBC, white blood cell; NEU, neutrophil; L, lymphocyte; mono, monocyte; RBC, red blood cell; P, platelet; CEA, carcinoembryonic antigen; CA199, carbohydrate antigen 199, CA724, carbohydrate antigen 724; CA125II, carbohydrate antigen 125II; IgA, immunoglobulin A; IgG, immunoglobulin G; KAP, light-chain immunoglobulin; LAM, heavy-chain immunoglobulin; SAT, subcutaneous fat area; VAT, visceral fat area.

When analyzing blood parameters, The one-way ANOVA and Kruskal-Wails rank-sum test found that SMI-IgM score was related to TP (total protein), ALB (albumin), PALB (prealbumin), L (lymphocyte), RBC (red blood cell), CEA (carcinoembryonic antigen), IgG, KAP (all *p* < 0.05). The detailed blood indicators of all 190 cases grouped by SMI-IgM score are displayed in **Table 1**. **Table 1** shows the detailed blood indicators of patients grouped by SMI-IgM score.

### Univariate and multivariate Cox’s regression analysis for PFS and OS

The univariate analysis found the patients’ age (*p* = 0.005), tumor size (*p*** < **0.001), pTNM stage (*p*** < **0.001), L (*p* = 0.021), CA724 (*p* = 0.002), IgG (*p* = 0.033), SAT (*p* = 0.020), VAT (*p* = 0.004), SMI-IgM score (*p*** < **0.001) were related to PFS. Our research indicates that the prognostic factors for patients with OS were age (*p* = 0.003), L (*p* = 0.017), tumor size (*p*** < **0.001), pTNM stage (*p*** < **0.001), CA724 (*p* = 0.002), IgG (*p* = 0.038), SAT (*p* = 0.020), VAT (*p* = 0.004), SMI-IgM score (*p*** < **0.001). The multivariate analysis indicated that CA724 (*p* = 0.015), pTNM stage (*p*** < **0.001), and SMI-IgM score (*p*** < **0.05) were independent prognostic factors for PFS. Our research findings suggest that the prognostic factors for patient OS were CA724 (*p* = 0.013), pTNM stage (*p*** < **0.001), and SMI-IgM score (*p*** < **0.05) (**Table 2**). We additionally performed supplementary analyses in which SMI and IgM were treated as continuous variables. In the univariate Cox regression analysis, SMI was significantly associated with both PFS (HR = 0.958, p = 0.014) and OS (HR = 0.960, p = 0.017), whereas IgM showed no significant association with PFS (HR = 0.580, p = 0.098) or OS (HR = 0.579, p = 0.098). We further assessed multicollinearity among the included predictors by calculating variance inflation factors (VIFs) (all <5), indicating no significant collinearity.

**Table 2:** Univariate and multivariate analysis for PFS and OS.

Parameters	PFS	P value	Multivariate analysis	P value	OS	P value	Multivariate analysis	P value
	Univariate analysis				Univariate analysis			
	Hazard ratio (95%CI)		Hazard ratio (95%CI)		Hazard ratio (95%CI)		Hazard ratio (95%CI)	
Sex (Male vs. Female)	0.929(0.540-1.597)	0.790			0.905(0.527-1.557)	0.719		
Age (<60 vs. ≥60)	2.220(1.280-3.849)	0.005	1.390(0.782-2.469)	0.262	2.284(1.317-3.960)	0.003	1.524(0.859-2.706)	0.150
BMI (22.07 kg/m2 vs. ≥22.07 kg/m2)	0.686(0.412-1.140)	0.146			0.692(0.416-1.150)	0.155		
Stomachache (NO vs. Yes)	1.710(0.890-3.285)	0.108			1.753(0.912-3.369)	0.092		
Melaena (NO vs. Yes)	1.555(0.897-2.696)	0.116			1.627(0.939-2.822)	0.083		
Weight loss (NO vs. Yes)	1.465(0.872-2.460)	0.149			1.478(0.880-2.482)	0.140		
Tumor size (<50 mm vs. ≥50 mm + unknown)	3.123(1.814-5.376)	<0.001	1.289(0.698-2.378)	0.418	3.099(1.800-5.333)	<0.001	1.296(0.704-2.385)	0.405
pTNM (0/Tis + I + II vs. III + IV)	7.053(4.076-12.206)	<0.001	4.891(2.637-9.072)	<0.001	6.618(3.831-11.431)	<0.001	4.946(2.670-9.160)	<0.001
ALT (<17 U/L vs. ≥17 U/L)	0.758(0.458-1.255	0.282			0.766(0.463-1.267)	0.299		
LDH (<160.5 U/L vs. ≥160.5 U/L)	1.441(0.869-2.389)	0.157			1.423(0.859-2.360)	0.171		
TBIL (<11.02 μmol/L vs. ≥11.02 μmol/L)	0.681(0.410-1.131)	0.138			0.663(0.399-1.103)	0.113		
TP (<68 g/L vs. ≥68 g/L)	1.043(0.631-1.723)	0.869			1.015(0.614-1.676)	0.955		
ALB (<41 g/L vs. ≥41 g/L)	0.817(0.494-1.351)	0.431			0.786(0.475-1.300)	0.348		
PALB (<264.5 mg/L vs. ≥264.5 mg/L)	0.630(0.378-1.050)	0.076			0.629(0.377-1.048)	0.075		
Urea (<5.65 mmol/L vs. ≥5.65 mmol/L)	1.173(0.709-1.941)	0.535			1.195(0.722-1.977)	0.489		
WBC (<6.39 109/L vs. ≥6.39 109/L)	0.787(0.475-1.304)	0.352			0.798(0.482-1.323)	0.383		
Neu (<3.60 109/L vs. ≥3.60 109/L)	0.978(0.592-1.616)	0.930			1.016(0.615-1.678)	0.951		
L (<1.90 109/L vs. ≥1.90 109/L)	0.542(0.323-0.910)	0.021	0.714(0.419-1.218)	0.216	0.532(0.317–0.894)	0.017	0.672(0.393-1.148)	0.146
Mono (<0.44 109/L vs. ≥0.44 109/L)	1.106(0.666-1.839)	0.696			1.136(0.683-1.887)	0.623		
RBC (<4.38 1012/L vs. ≥4.38 1012/L)	0.661(0.397-1.102)	0.113			0.660(0.396-1.099)	0.110		
P (<252 109/L vs. ≥252 109/L)	1.022(0.619-1.689)	0.932			1.071(0.648-1.769)	0.790		
CEA (<1.98 ng/mL vs. ≥1.98 ng/mL)	1.444(0.869-2.389)	0.156			1.547(0.931-2.572)	0.092		
CA199 (<9.43 U/mL vs. ≥9.43 U/mL)	1.613(0.968-2.688)	0.066			1.642(0.985-2.737)	0.057		
CA724 (<2.10 U/mL vs. ≥2.10 U/mL)	2.277(1.342-3.863)	0.002	1.998(1.143-3.491)	0.015	2.274(1.340-3.859)	0.002	2.046(1.166-3.590)	0.013
CA125II (<9.80 U/mL vs. ≥9.80 U/mL)	1.641(0.985-2.734)	0.057			1.613(0.938-2.687)	0.067		
IgA (<2.22 g/L vs. ≥2.22 g/L)	1.409(0.787-2.525)	0.249			1.375(0.768-2.464)	0.284		
IgG (<10.70 g/L vs. ≥10.70 g/L)	0.563(0.332-0.955)	0.033	1.221(0.693-2.149)	0.490	0.571(0.337-0.969)	0.038	1.301(0.738-2.295)	0.363
KAP (<8.33 g/L vs. ≥8.33 g/L)	1.001(0.313-3.199)	0.998			1.020(0.319-3.257)	0.974		
LAM (<4.71 g/L vs. ≥4.71 g/L)	0.732(0.389-1.376)	0.332			0.731(0.389-1.375)	0.331		
KAP/LAM (<1.78 vs. ≥1.78)	0.680(0.411-1.126)	0.134			0.710(0.429-1.176)	0.183		
SAT (<119.73 cm² vs. ≥119.73 cm²)	0.394(0.179-0.865)	0.020	1.247(0.503-3.092)	0.633	0.392(0.178-0.861)	0.020	1.169(0.476-2.869)	0.734
VAT (<100.14 cm² vs. ≥100.14 cm²)	0.288(0.124-0.669)	0.004	0.426(0.164-1.107)	0.080	0.287(0.124-0.668)	0.004	0.451(0.174-1.169)	0.101
SMI-IgM score								
score 1	Ref		Ref		Ref		Ref	
score 2	0.406(0.240-0.686)	0.001	0.535(0.307-0.930)	0.027	0.409(0.242-691)	0.001	0.493(0.285-0.855)	0.012
score 3	0.073(0.018-0.305)	<0.001	0.149(0.034-0.659)	0.012	0.072(0.017-0.301)	<0.001	0.127(0.029-0.553)	0.006

BMI, body mass index; ALT, alanine aminotransferase; LDH, lactate dehydrogenase; TBIL, total bilirubin; TP, total protein; ALB, albumin; PALB, prealbumin; WBC, white blood cell; NEU, neutrophil; L, lymphocyte; mono, monocyte; RBC, red blood cell; P, platelet; CEA, carcinoembryonic antigen; CA199, carbohydrate antigen 199, CA724, carbohydrate antigen 724; CA125II, carbohydrate antigen 125II; IgA, immunoglobulin A; IgG, immunoglobulin G; KAP, light-chain immunoglobulin; LAM, heavy-chain immunoglobulin; SAT, subcutaneous fat area; VAT, visceral fat area.

### Survival analysis for IgM and SMI

**Figure 2:**
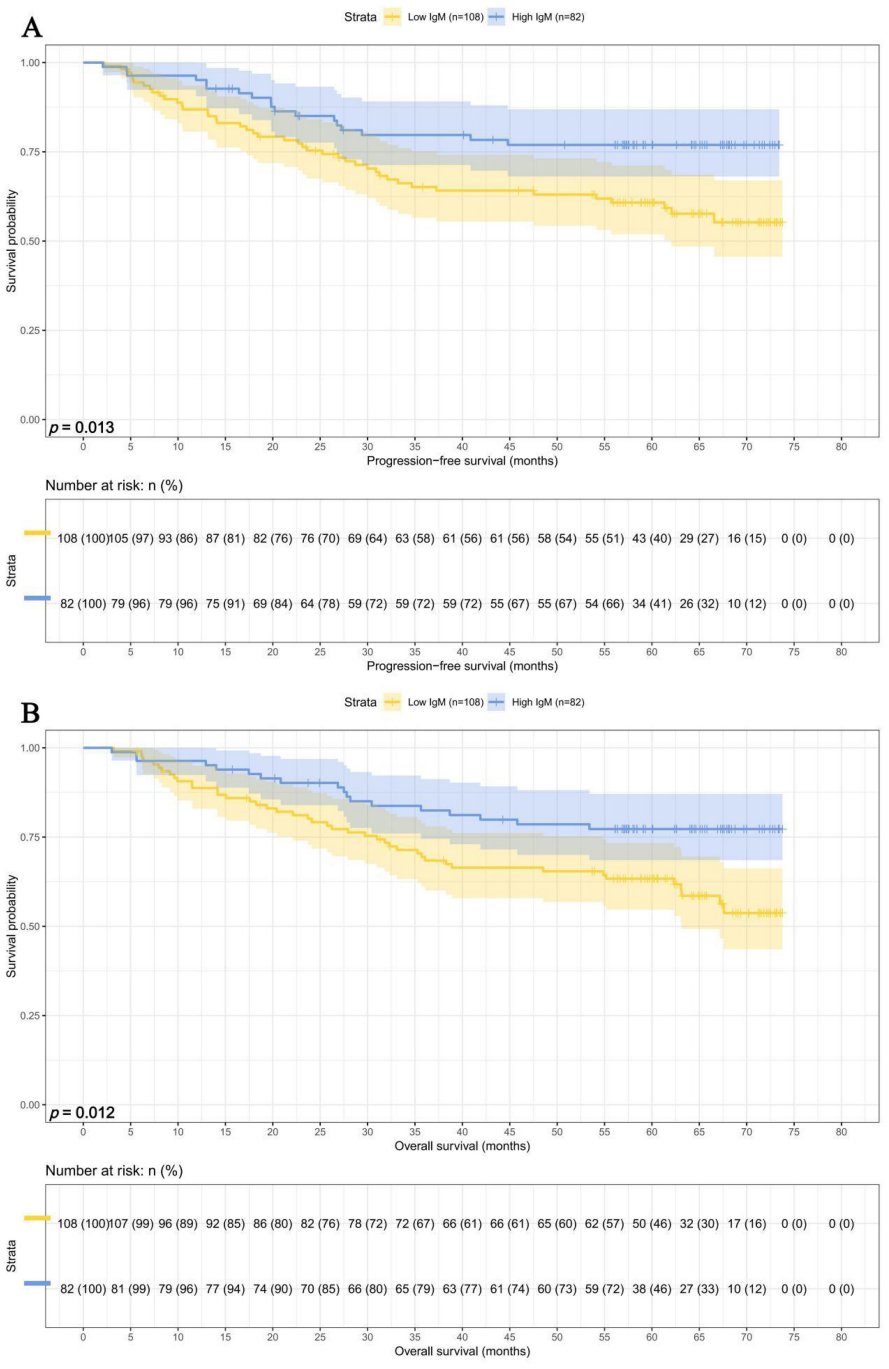
IgM related survival curve of **(A)** PFS and **(B)** OS in all patients.

In this study, we performed survival analyses for IgM and SMI separately. Among the 108 patients with IgM < 0.93 g/L, the 1-, 3-, and 5-year survival rates were 86.9% (95% CI: 80.7%-93.5%), 65.2% (95% CI:56.6%-75.1%), and 60.8% (95% CI: 51.9%-71.1%) for PFS, and 88.7% (95% CI: 82.9%-95.0%), 69.4% (95% CI: 61.1%-78.9%), and 63.3% (95% CI: 54.7%-73.4%) for OS. In contrast, among the 82 patients with IgM ≥ 0.93 g/L, the corresponding survival rates were 95.1% (95% CI: 90.6%-99.9%), 79.7% (95% CI: 71.3%-89.1%), and 76.9% (95% CI: 68.1%-86.9%) for PFS, and 96.3% (95% CI: 92.4%-100.0%), 82.4% (95% CI: 74.5%-91.2%), and 77.3% (95% CI: 68.5%-87.1%) for OS. Patients with higher IgM levels exhibited prolonged PFS (HR = 0.498, p = 0.013) and OS (HR = 0.493, p = 0.012) (**Figures 2A, B**).

**Figure 3:**
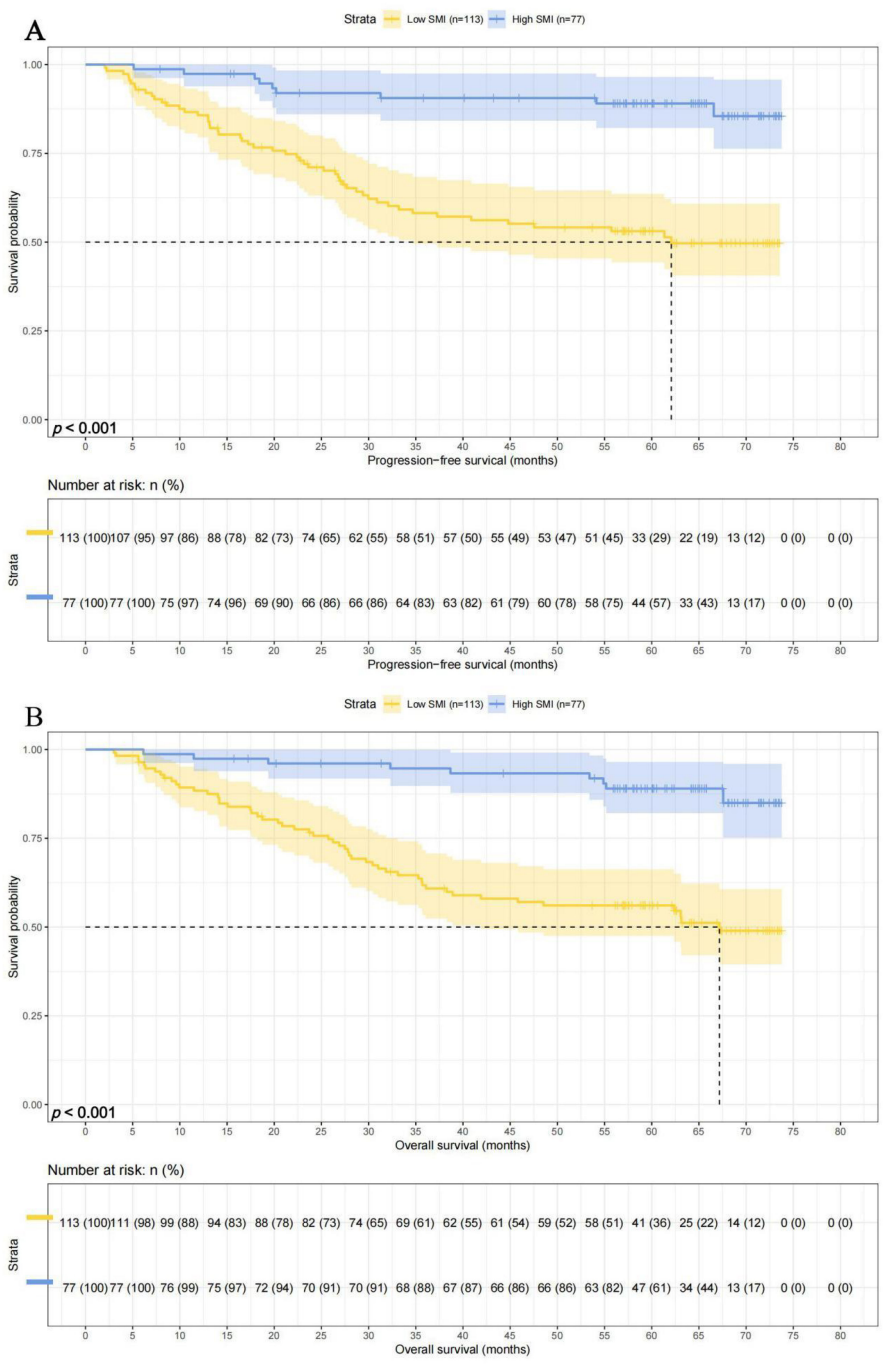
SMI related survival curve of **(A)** PFS and **(B)** OS in all patients.

There were 113 patients with lower SMI, and their 1-, 3-, and 5-year survival rates for PFS and OS were 85.7% (95% CI: 79.5%-92.5%), 58.2% (95% CI: 49.5%-68.4%), and 53.1% (95% CI: 44.3%-63.6%) and 88.4% (95% CI: 82.6%-94.5%), 61.8% (95% CI: 53.3%-71.6%),and 51.6% (95% CI: 47.5%-66.3%), respectively. While there were 73 patients with higher SMI, and their 1-, 3-, and 5-year survival rates for PFS and OS were 97.4% (95% CI: 93.9%-100.0%), 90.6% (95% CI: 84.2%-97.5%), and 89.0% (95% CI: 82.1%-96.5%) and 97.4% (95% CI: 93.9%-100.0%), 94.7% (95% CI: 89.7%-99.9%), and 89.0% (95% CI: 82.1%-96.5%), respectively. Patients with high SMI levels had longer PFS (HR = 0.196, p < 0.001) and OS (HR = 0.195, p < 0.001) (**Figures 3A, B**).

### SMI-IgM score and prognosis

**Figure 4:**
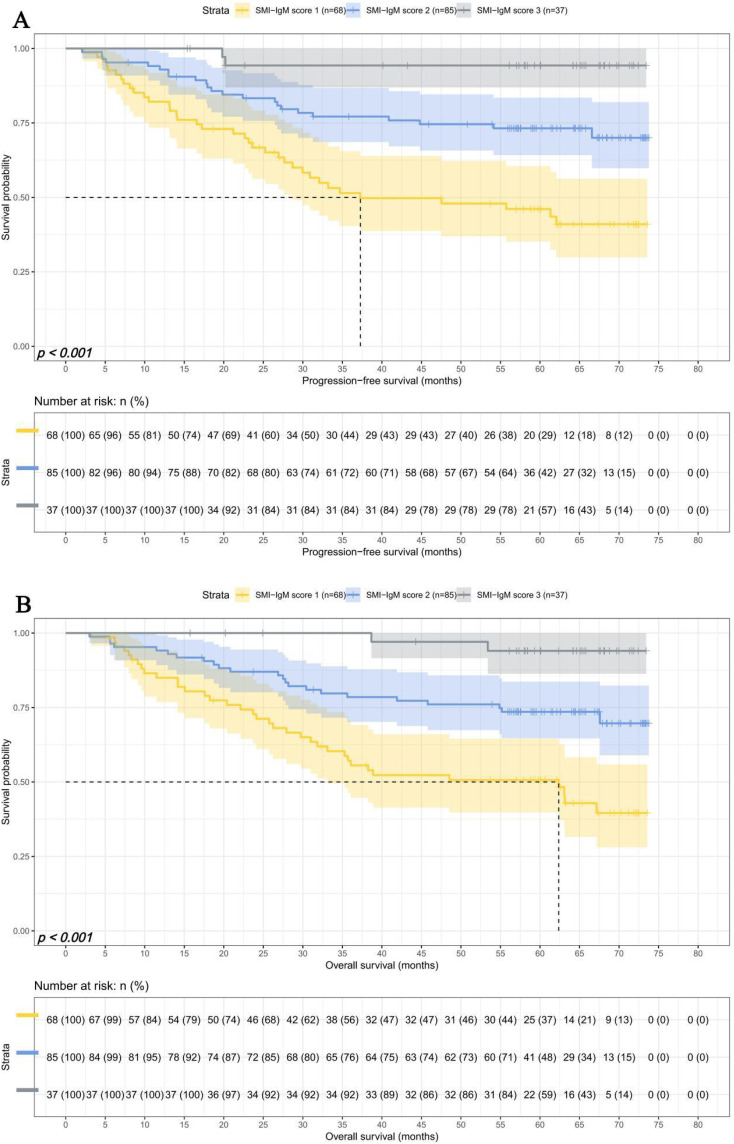
SMI-IgM score related survival curve of **(A)** PFS and **(B)** OS in all patients.

The median survival times for PFS and OS in SMI-IgM score 1 group were 37.27 months and 62.37 months. The 1-, 3-, and 5- year survival rates for PFS in SMI-IgM score 1 group were 82.1% (95% CI: 73.4%-91.8%), 51.4% (95% CI: 40.4%-65.5%), 46.1% (95% CI: 35.1%-60.5%). The 1-, 3-, and 5- year survival rates for OS in SMI-IgM score 1 were 85.0% (95% CI: 76.8%-94.0%), 57.2% (95% CI: 46.3%-70.6%), 50.7% (95% CI: 39.8%-64.5%). The median survival time of PFS in SMI-IgM score groups 2 and 3 were both not achieved. The 1-, 3-, and 5-year survival rates for PFS in SMI-IgM score groups 2 and 3 were 92.9% (95% CI: 87.6%-98.5%), 77.1% (95% CI: 68.6%-86.7%), and 73.2% (95% CI: 64.2%-83.5%); 100.0% (95% CI: 100.0%-100.0%), 94.3% (95% CI: 86.9%-100.0%), and 94.3% (95% CI: 86.9%-100.0%), respectively. The median survival time of OS in SMI-IgM score groups 2 and 3 were not achieved. The 1-, 3-, and 5-year survival rates for OS in SMI-IgM score groups 2 and 3 were 94.1% (95% CI: 89.2%-99.3%), 78.5% (95% CI: 70.2%-87.8%), and 73.6% (95% CI: 64.6%-83.7%); 100.0% (95% CI: 100.0%-100.0%), 100.0% (95% CI: 100.0%-100.0%), and 94.0% (95% CI: 86.3%-100.0%), respectively. Patients with SMI-IgM score 1 had worse PFS (HR = 0.345, 95% CI: 0.226-0.525, *p*** < **0.001) and OS (HR = 0.345, 95% CI: 0.227-0.525, *p*** < **0.001) (**Figures 4A, B**).

### Survival for pTNM stage

**Figure 5:**
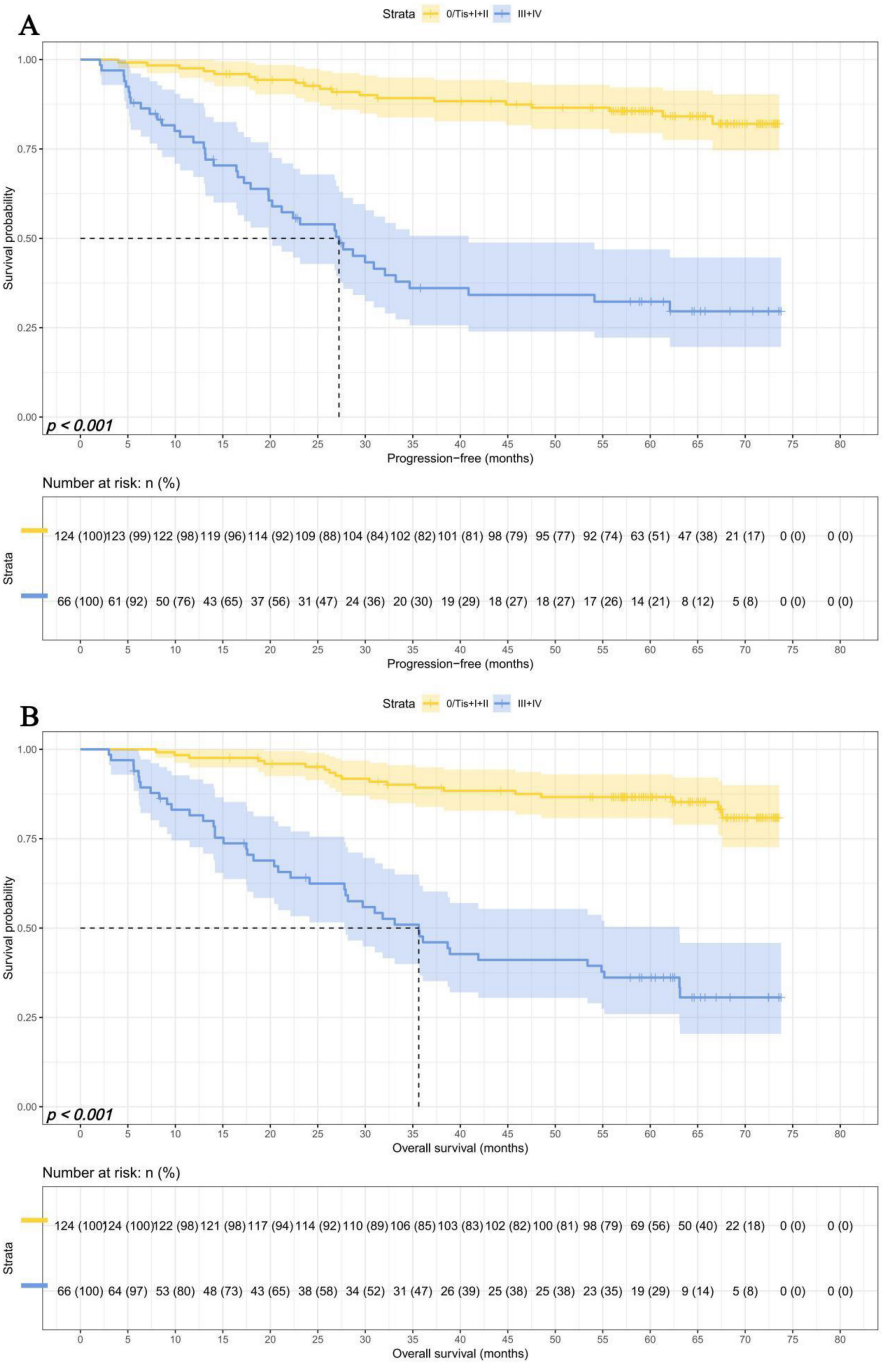
The pTNM related survival curve of **(A)** PFS and **(B)** OS in all patients.

Due to differences in TNM staging among patients, we investigated the predictive ability of SMI-IgM score and the prognostic significance of pTNM staging. We divided 190 patients into an early pTNM stage (0/Tis + I + II) group (124 patients) and an advanced pTNM stage (III + IV) group (66 patients). The median survival time for PFS and OS in early pTNM stage and advanced pTNM stage were both not reached. The 1-, 3-, and 5-year survival rates for PFS in early pTNM are 97.6% (95% CI: 94.9%-100.0%), 89.2% (95% CI: 83.8%-94.9%), and 85.6% (95% CI: 79.5%-92.2%). The 1-, 3-, and 5-year survival rates for OS in early pTNM are 97.6% (95% CI: 94.9%-100.0%), 89.2% (95% CI: 83.9%-94.9%), and 86.7% (95% CI: 80.8%-93.0%). The median survival time for PFS and OS in advanced pTNM stage and advanced pTNM stage were 26.93 months and 35.63 months. The 1-, 3-, and 5-year survival rates for PFS and OS in early pTNM were 76.8% (95% CI: 67.2%-87.8%), 36.1% (95% CI: 25.7%-50.7%), and 32.3% (95% CI: 22.2%-46.9%); 81.5% (95% CI: 72.6%-91.5%), 47.7% (95% CI: 36.7%-61.8%), 36.1% (95% CI: 25.9%-50.4%), respectively. Patients in the advanced pTNM stage had lower PFS (HR = 6.983, 95% CI: 4.027-12.108, *p*** < **0.001) and OS (HR = 6.618, 95% CI: 3.831-11.431, *p*** < **0.001) than those early pTNM stage patients (**Figures 5A, B**).

**Figure 6:**
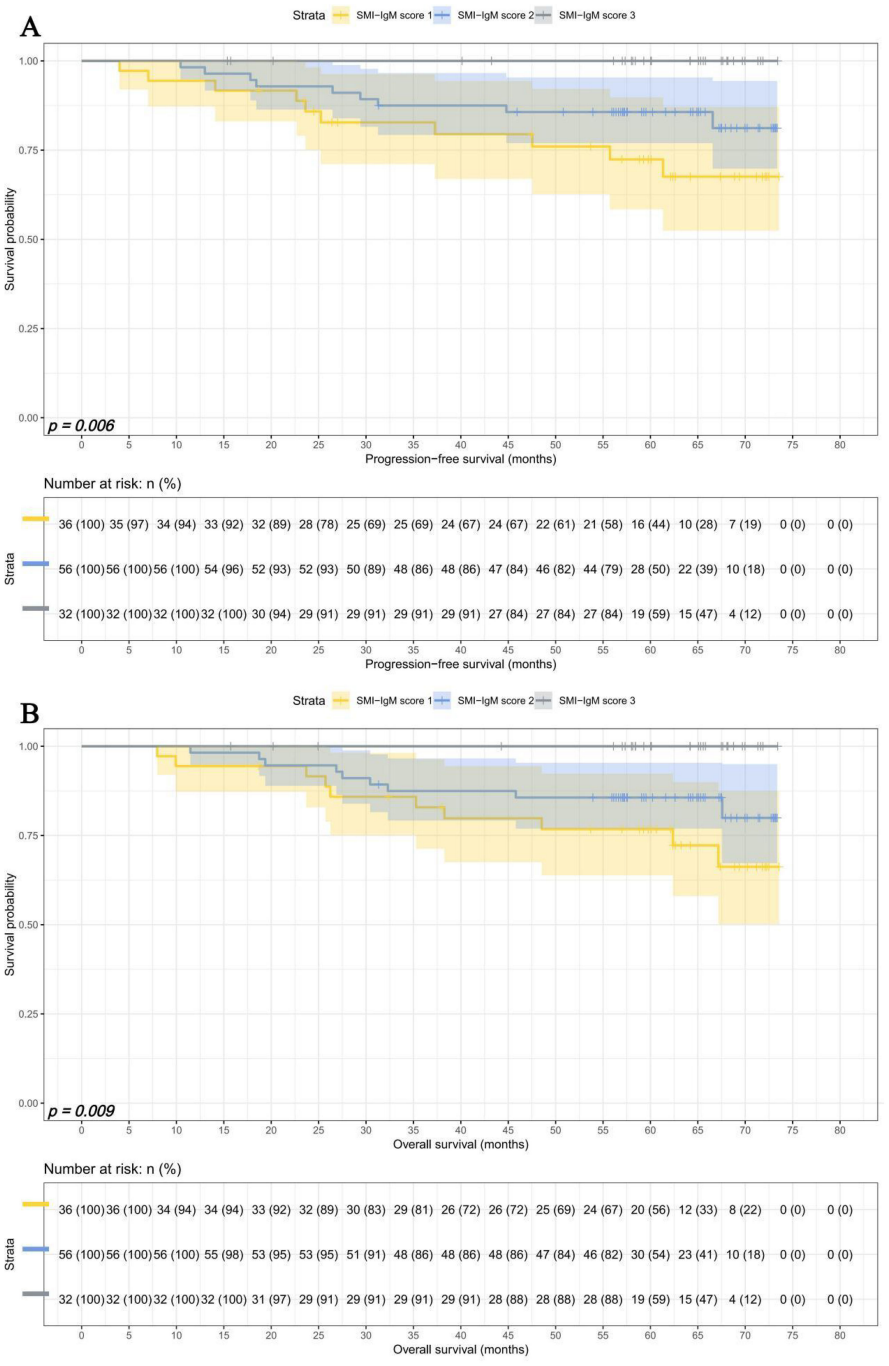
SMI-IgM score related survival curves in early pTNM stage for **(A)** PFS and **(B)** OS.

In early pTNM stage, There were 36 patients in SMI-IgM score 1 group, 56 patients in SMI-IgM score 2 group, and 32 patients in SMI-IgM score 3 group. The 1-, 3-, and 5-year survival rates for PFS and OS in SMI-IgM score 1 were 94.4% (95% CI: 87.3%-100.0%) vs. 94.4% (95% CI: 87.3%-100.0%), 82.8% (95% CI: 71.1%-96.4%) vs. 82.9% (95% CI: 71.3%-96.4%), and 72.4% (95% CI: 58.4%-89.8%) vs. 76.8% (95% CI: 63.8%-92.3%). The 1-, 3-, and 5-year survival rates for PFS and OS in SMI-IgM score 2 were 98.2% (95% CI: 94.8%-100.0%), 87.5% (95% CI: 79.3%-96.6%), and 85.7% (95% CI: 77.0%-95.4%); 98.2% (95% CI: 94.8%-100.0%), 87.5% (95% CI: 79.2%-96.6%), and 85.6% (95% CI: 76.9%-95.4%), respectively. The 1-, 3-, and 5-year survival rates for PFS and OS in SMI-IgM score 3 were 100.0% (95% CI: 100.0%-100.0%) vs. 100.0% (95% CI: 100.0%-100.0%), 100.0% (95% CI: 100.0%-100.0%) vs. 100.0% (95% CI: 100.0%-100.0%), and 100.0% (95% CI: 100.0%-100.0%) vs.100.0% (95% CI: 100.0%-100.0%). Patients with SMI-IgM score 1 had shorter PFS (HR = 0.325, 95% CI: 0.156-0.675, *p* = 0.003) and OS (HR = 0.342, 95% CI: 0.166-0.708, *p* = 0.004) (**Figures 6A, B**).

**Figure 7:**
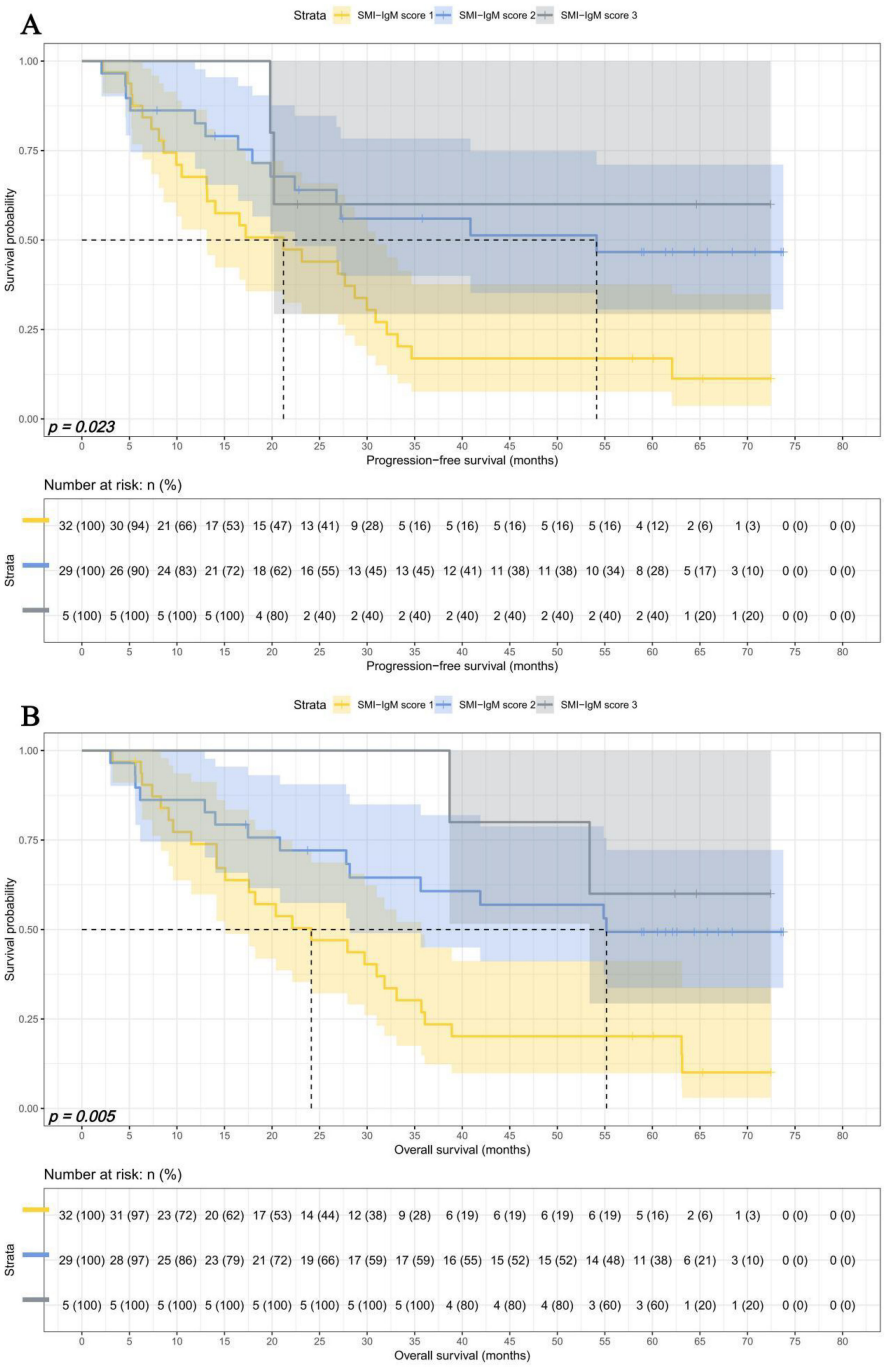
SMI-IgM score related survival curves in advanced pTNM stage for **(A)** PFS and **(B)** OS.

In advanced pTNM stage, There were 32 patients in SMI-IgM score 1 group, 29 patients in SMI-IgM score 2 group, and 5 patients in SMI-IgM score 3 group. The 1-, 3-, and 5-year survival rates for PFS and OS in SMI-IgM score 1 were 67.6% (95% CI: 52.9%-86.4%) vs. 73.9% (95% CI: 59.8%-91.2%), 16.9% (95% CI: 7.6%-37.5%) vs.26.9% (95% CI: 14.9%-48.5%), and 16.9% (95% CI: 7.6%-37.5%) vs. 20.2% (95% CI: 9.9%-41.2%). The 1-, 3-, and 5-year survival rates for PFS and OS in SMI-IgM score 2 were 82.6% (95% CI: 69.9%-97.7%), 56.0% (95% CI: 40.0%-78.3%), and 46.6% (95% CI: 30.6%-71.1%); 86.2% (95% CI: 74.5%-99.7%), 60.7% (95% CI: 45.0%-81.9%), and 49.3% (95% CI: 33.7%-72.2%), respectively. The 1-, 3-, and 5-year survival rates for PFS and OS in SMI-IgM score 3 were 100.0% (95% CI: 100.0%-100.0%) vs. 100.0% (95% CI: 100.0%-100.0%), 60.0% (95% CI: 29.3%-100.0%) vs. 100.0% (95% CI: 100.0%-100.0%), and 60.0% (95% CI: 29.3%-100.0%) vs. 60.0% (95% CI: 29.3%-100.0%). Patients with SMI-IgM score 1 had worse PFS (HR = 0.491, 95% CI: 0.284-0.850, *p* = 0.011) and OS (HR = 0.434, 95% CI: 0.252-0.746, *p* = 0.003) (**Figures 7A, B**).

### Subgroup analysis

**Figure 8:**
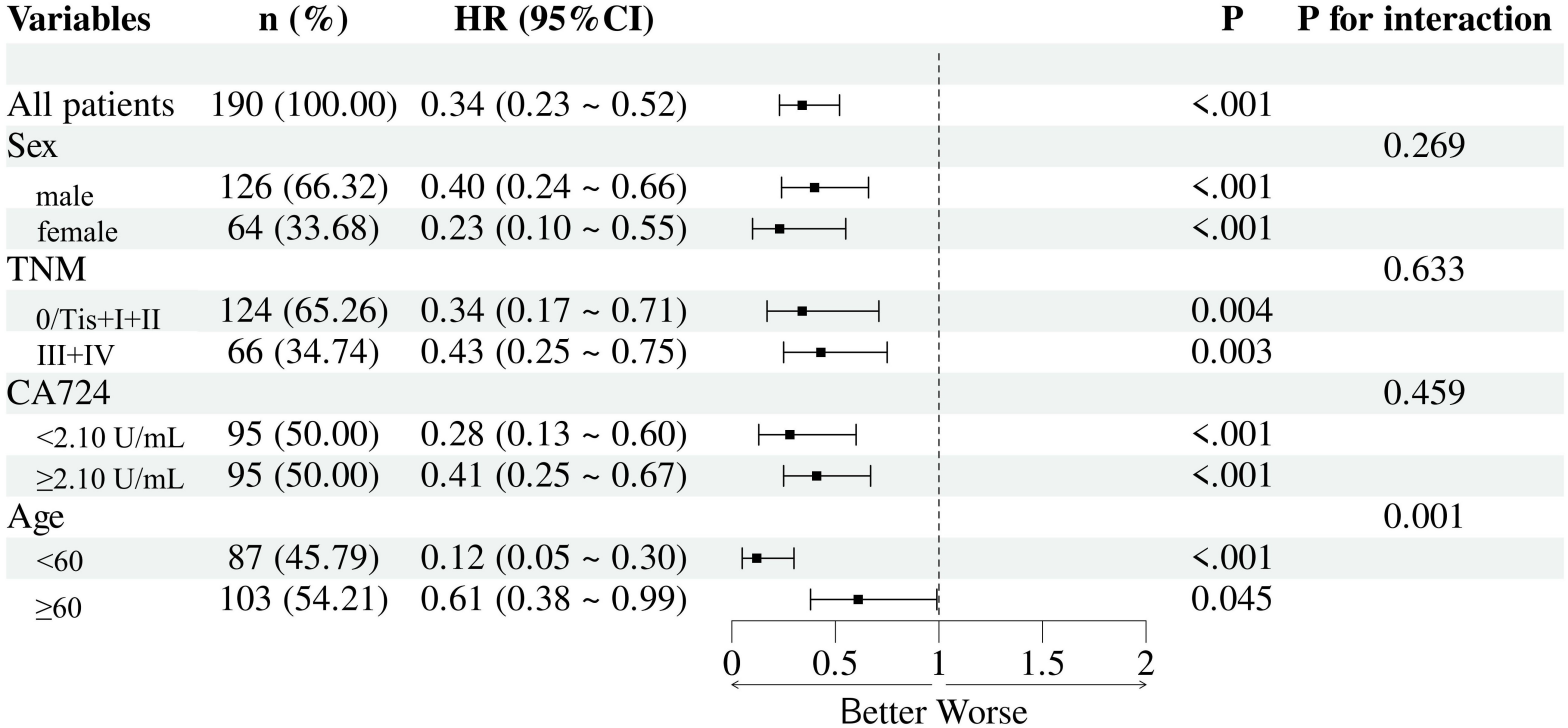
The stratification analysis of SMI-IgM score for OS.

Subgroup analyses were conducted to assess the prognostic value of the SMI-IgM across different clinical strata, including sex, TNM stage, CA724 level, and age. The SMI-IgM remained a significant predictor of overall survival in most subgroups, with no significant interactions observed for sex, TNM stage, or CA724 level. Notably, a significant interaction was detected for age, indicating that the prognostic impact of the SMI-IgM index was more pronounced in younger patients (**Figure 8**).

### Construction of nomograms to predict PFS and OS

**Figure 9:**
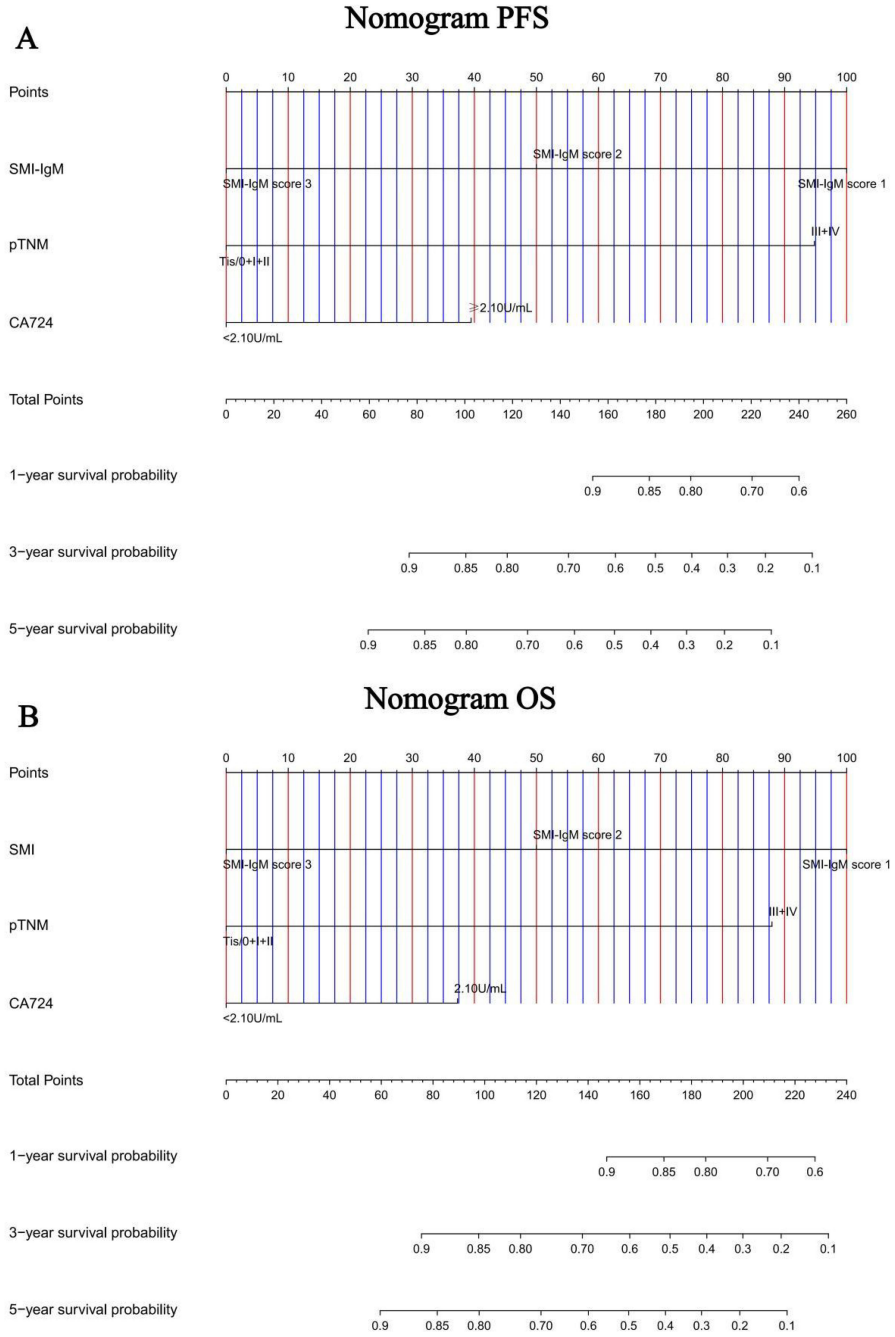
Nomogram for predicting 1-, 3-, 5-year survival probability of **(A)** PFS and **(B)** OS.

**Figure 10:**
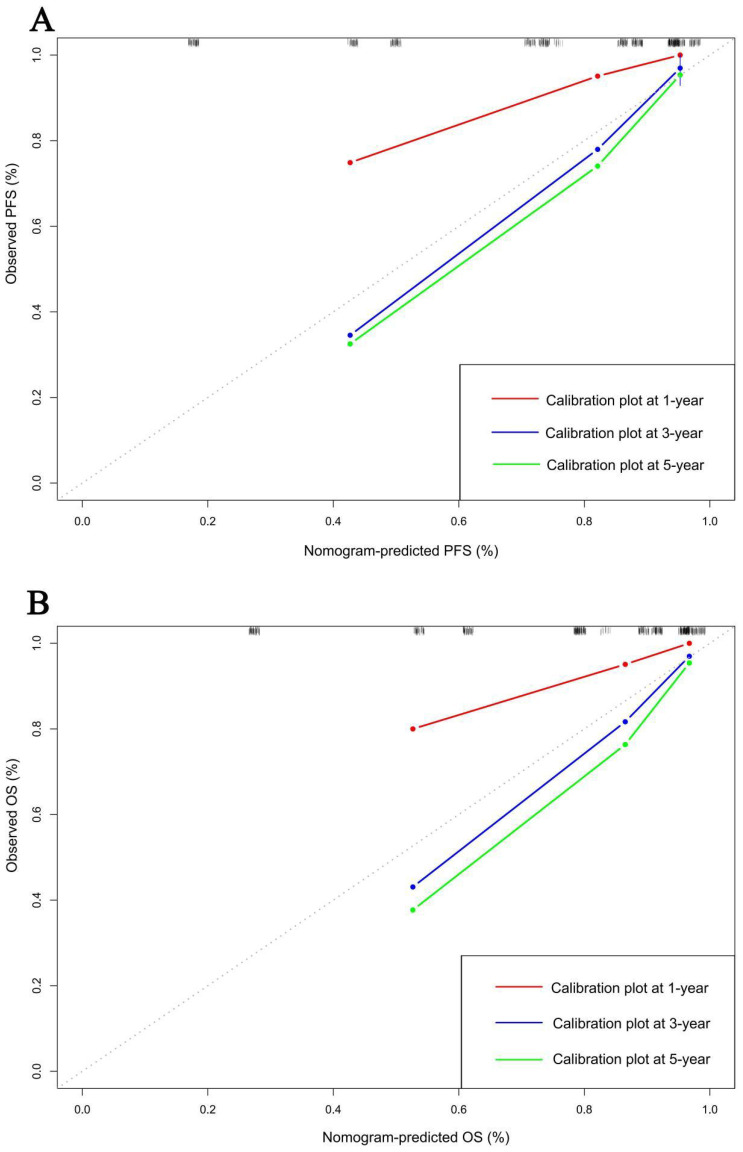
Calibration curves for predicting **(A)** PFS and **(B)** OS at 1-,3-, and 5-years.

In order to further test the prognostic effectiveness of SMI-IgM score, we constructed nomograms based on CA724, pTNM stage, SMI-IgM score to predict the 1-, 3-, and 5- year survival probability for PFS and OS. The C-index and 95% CI for predicting the survival probability of PFS and OS were 0.808 (0.761-0.855) and 0.806 (0.758-0.854), respectively (**Figures 9A, B**). The calibration found that the nomograms could accurately predict the 3- and 5-year survival rates of PFS and OS in patients (**Figures 10A, B**). To evaluate the internal robustness and discrimination performance of the prognostic models, bootstrap resampling with 1000 iterations was conducted. The optimism-corrected concordance index was 0.802 for PFS and 0.801 for OS, suggesting that both models demonstrated strong predictive discrimination with minimal overfitting.

## Discussion

Our study is the first to assess the relationship between SMI-IgM score and the prognosis of patients who underwent surgery for gastric cancer. Our results showed that SMI-IgM score was an independent prognostic factor for PFS and OS in patients underwent radical gastric cancer surgery. In addition we found that pTNM stage and CA724 were an independent prognostic factor for PFS and OS. This study suggests that low SMI and low IgM are associated with a poorer prognosis for patients.

Gastric cancer is one of the most common malignant tumors in China, with the third highest incidence and mortality rates in China (26). Nowadays, there are various therapeutic methods for gastric cancer, including surgery, chemotherapy, immunotherapy, and so on (2, 27, 28). The survival rate of gastric cancer patients has been greatly improved, but there are still many patients with poor prognosis (29). Previous studies have shown that the prognosis of gastric cancer patients is related to the nutritional status and immune function of the organism (30–32).

Although a series of studies have shown that SMI and IgM can predict recurrent metastasis in gastric cancer (33, 34) and other malignancies (35–37). However, there are no articles that combine SMI and IgM to form the SMI-IgM score to predict progression in patients underwent surgery for gastric cancer. In our study, low SMI and low IgM were associated with poorer prognosis in patients with gastric cancer. The SMI-IgM score could predict the prognosis of patients with gastric cancer, which may be explained by the following mechanisms. SMI is an accurate indicator for assessing the nutritional status of the body in relation to sarcopenia. Elderly people and patients with malignant tumors are prone to reduced SMI, which is an important cause of frailty (functional limitations and physical disabilities), the extent of which affects the body’s immune function (38). Several studies have shown that muscle loss may lead to immune senescence. Skeletal muscle cells secrete large amounts of interleukin 15 (IL-15), which is important for natural killer (NK) cell development. NK cells have an important role in tumor killing (39, 40). Skeletal muscle loss may alter immune cell populations such as myeloid-derived suppressor cells via myocytokines (41, 42). Skeletal muscle loss can be affected by inflammation in the body (43). Cancer is a chronic inflammatory disease, and inflammatory cells such as neutrophils produced by the cancer stimulus can progress the tumor by invading adipose tissue and causing the body to become depleted of nutrients (44). IgM mainly reflects the state of the body’s recent immune response (45). Tumor-reactive IgM could clear tumor cells through complement fixation, induction of apoptosis and induction of secondary immune responses against neoantigens (46–48). Serum WT1–271 IgM antibodies exhibit high sensitivity for early-stage gastric cancer and can serve as a diagnostic marker for gastric cancer when combined with autoantibody screening, especially in the early stages (49). IgM SC-1 recognizes a tumor-specific carbohydrate epitope on decay-accelerating factor B (DAF; also known as CD55), which is selectively expressed on the membrane of gastric carcinoma cells, and induces apoptosis through receptor crosslinking both *in vitro* and in experimental *in vivo* models. IgM PAM-1 targets CFr-1 (cysteine-rich fibroblast growth factor receptor), and its binding inhibits growth factor receptor pathways such as EGFR and FGFR, which are frequently overexpressed in malignant cells, ultimately leading to cellular starvation and death (50, 51).

A study involving 1,516 patients showed that body mass index was negatively associated with IgM concentrations after adjusting for covariates (52). Moreover, gastric cancer patients who received immune-enhanced enteral nutrition showed significant increases in IgM, NK cell, and albumin levels (53, 54), indicating that IgM levels not only reflect recent immune responses but are also influenced by the patient’s current nutritional status and degree of obesity. However, IgM levels are also affected by non-cancer-related nutritional and immune conditions, such as infections caused by recent pathogens and autoimmune diseases. Therefore, its application in cancer research and clinical practice is limited (55). SMI reflects the long-term nutritional, immune, and inflammatory status of gastric cancer patients, whereas IgM is more closely associated with recent immune and nutritional conditions. Combining these two indicators allows for a multidimensional assessment of the patient’s physiological status during cancer progression, thereby providing a basis for prognosis evaluation and nutritional support therapy in gastric cancer patients. The coexistence of low SMI and low IgM (SMI-IgM score 1) may synergistically impair both nutritional and immune defense systems, leading to worse outcomes, while discordant cases (score 2) may reflect partial compensation. In our study, while pTNM stage and CA724 remained the strongest predictors, the SMI-IgM score provided complementary information reflecting the nutritional and immune status, which were not captured by tumor-based factors alone.

This study, while providing valuable insights, is not without its limitations. Firstly, it is imperative to acknowledge that this was a single-region, single-center retrospective investigation characterized by a relatively modest sample size, which may introduce inherent biases. Furthermore, the study exclusively focused on gastric cancer patients who had undergone surgical intervention, potentially limiting the generalizability of the findings. We acknowledge that results may differ in non-surgical or advanced-stage patients and emphasize that further studies are required to validate generalizability. Additionally, the determination of SMI and IgM cut-off values was reliant on ROC curves, and it is worth noting that these optimal cut-off values exhibited regional variations. Normalization or consensus-based cut-offs may improve reproducibility across populations. Because of the retrospective design of our study, detailed data regarding postoperative chemotherapy regimens, perioperative complications, and comorbidities were incomplete and therefore could not be reliably included in the multivariate analyses. Consequently, there is a compelling need for prospective clinical trials encompassing more extensive sample sizes and multiple geographical regions, involving diverse medical centers, to robustly validate these findings.

## Conclusion

In our study, we found that SMI-IgM score was an independent predictor of PFS and OS. This novel index demonstrates efficacy in prognosticating the recurrence and metastasis risk in gastric cancer patients who subjected to surgical interventions. As elucidated, a lower SMI-IgM score aligns with a deteriorating prognosis, substantiating its utility as a novel predictive tool in the selection and management of gastric cancer patients underwent surgical interventions.

## Data Availability

The raw data supporting the conclusions of this article will be made available by the authors, without undue reservation.
